# Relationship Gender Equality and Couples' Uptake of Oral Human Immunodeficiency Virus Self-Testing Kits Delivered by Pregnant Women in Kenya

**DOI:** 10.1097/OLQ.0000000000001037

**Published:** 2019-07-23

**Authors:** Caroline J. Vrana-Diaz, Jeffrey E. Korte, Mulugeta Gebregziabher, Lauren Richey, Anbesaw Selassie, Michael Sweat, Anthony Gichangi

**Affiliations:** From the ∗ Department of Public Health Sciences, Medical University of South Carolina, Charleston, SC; † Section of Infectious Disease, Department of Medicine, Louisiana State University Health Sciences Center, New Orleans, LA; ‡ Department of Psychiatry and Behavioral Sciences, Medical University of South Carolina, Charleston, SC; § Jhpiego, Ring Rd, Arlington block, Nairobi, Kenya

## Abstract

**Background:**

Higher gender equality is associated with many human immunodeficiency virus (HIV) preventive behaviors, including HIV testing. HIV self-testing is a relatively new testing technology that could assist with HIV prevention. However, there are no studies examining gender equality and HIV self-testing. We examined the associations between gender equality and couples' uptake of HIV self-testing among heterosexual couples expecting a child in central Kenya.

**Methods:**

This analysis used data from a HIV self-testing randomized intervention trial among pregnant women attending antenatal care and their male partners. The primary exposures were gender equality (measured by the male partner's attitudes toward intimate partner violence, and the woman's report on her household decision making power), and the primary outcome was couples' uptake of HIV self-testing. Generalized linear mixed models framework was used to account for site-level clustering.

**Results:**

In comparison to male partners reporting high acceptance of intimate partner violence, couples with male partners reporting medium acceptance (odds ratio, 2.36; 95% confidence interval, 0.99–5.63) or low acceptance (odds ratio, 2.50; 95% confidence interval, 1.20–5.21) were significantly more likely to use HIV self-testing. Gender equality measured by decision making power was not associated with couples' uptake of HIV self-testing.

**Conclusions:**

This study is the first of its kind to examine the association between gender equality and couples' HIV self-testing. This holds important implications for HIV self-testing as we strive to achieve the United Nations Programme on HIV/acquired immune deficiency syndrome goal that 90% of individuals living with HIV should know their status.

## Abstract 2

A study of pregnant couples in Kenya found that male partners with low acceptance of intimate partner violence were more likely to use human immunodeficiency virus self-testing than those with high acceptance of intimate partner violence.

Higher gender equality, especially within sexual relationships, has been associated with many human immunodeficiency virus (HIV) preventive behaviors, including condom use, reduced partner concurrency, use of preexposure prophylaxis, use of HIV antiretroviral treatment, and decreased involvement in other sexually risky behaviors.^1–9^ One important HIV preventive behavior is HIV testing. A literature review assessing gender equality and sexual and reproductive health found that women who face violence within their relationship were less likely to access HIV testing services.^10^ A study focusing on married women in Kenya, Zimbabwe, and Zambia found that education (a key element of gender equality) was positively associated with married women testing for HIV and found that the belief that gender-based violence is never acceptable (a key gender equality measure) was positively associated with older married women testing for HIV, and high financial decision making (another key gender equality measure) was positively associated with HIV testing for older married women in Zimbabwe.^11^

However, these studies were assessing HIV testing, traditionally accomplished in Kenya by use of rapid blood tests in health clinics. A new method of HIV testing is HIV self-testing (HST), which has been established as a potential alternative to or preliminary method before clinic-based testing. In 2012, the United States Food and Drug Administration approved the OraQuick In-Home HIV Test as the first rapid HIV self-test to be purchased over the counter in the United States.^12^ The HST has been shown to be a discreet and convenient method of testing that can reduce barriers to conventional HIV testing and has high acceptability, feasibility, and accuracy among many different populations.^13,14^ The World Health Organization gave a recommendation that there is moderate quality evidence for HST, and they put forth a strong recommendation that HST should be offered as an additional approach to HIV testing services.^15^ The Kenyan Ministry of Health made HIV self-test kits available at both public and private health facilities and selected pharmacies in May of 2017.^16^ Studies involving behavioral change interventions regarding HIV prevention within couples have also been shown to reduce HIV transmission among serodiscordant couples.^17–19^ In sub-Saharan Africa, the majority of new infections are from heterosexual transmission, so testing among heterosexual couples is extremely important.^20^ From 2013 to 2015, the percentage of women who tested for HIV while pregnant grew from 56% to 93%, whereas the HIV mother-to-child transmission rates decreased from 14% to 8.3% in the same timeframe.^21^ Sixty percent of those new infections among children in 2015 were from mothers diagnosed late in pregnancy or while attending postnatal services.^21^ This shows the importance of both members of a pregnant partnership testing for HIV early to help prevent mother-to-child transmission of HIV. The National AIDS and STI Control Programme in Kenya has recommended couples testing due to these important benefits.^22^ Therefore, HST could be used not only to improve testing rates in individuals, but also to improve the rates of couples testing for HIV.

This is the first study of its kind to study gender equality and how it is associated with couples' uptake of HST. To address this question, we used data from a randomized controlled trial of an HST intervention among heterosexual couples expecting a child in central Kenya. Our aim was to identify the associations between gender equality (measured by decision making power and attitudes toward intimate partner violence [IPV]) and uptake of the couples' HST. We hypothesized that higher gender equality will be associated with higher uptake of HST by couples.

## METHODS

### Design and Study Population

This analysis uses data from a HIV self-testing randomized intervention trial that was conducted in 14 separate clinics within 5 counties in central and eastern Kenya between July 2015 and February 2016.^23^ The primary objective of this trial was to assess the impact of HST on male partner HIV testing rates. Women could participate in the study if they were at least 18 years old and pregnant, and attending antenatal clinic (ANC) for the first time in this pregnancy. The women also had to have reported contact with their male partner (not necessarily the father of the child) at least once per week, believe their male partner was either HIV-negative or had unknown status at recruitment, and report that their male partner had not tested for HIV in the past 3 months before the study. If the women were concerned about a potential for violence from their male partner due to the topic of HIV testing, they were excluded due to safety concerns, but very few women were excluded for this reason. Women were randomized into 1 of 3 arms after providing informed consent and completing a baseline questionnaire. Arm 1 was based on the standard Kenyan Ministry of Health card that invites the male partner to come to the health clinic for a discussion on family health but did not mention HIV in the card. Arm 2 included an enhanced invitation card that described not only family health, but the benefits of the male partner testing for HIV to prevent mother-to-child transmission of HIV. Arm 3 included the card from Arm 2 plus two OraQuick HST kits with instructions to test for HIV at home. It is standard to test the woman for HIV as part of ANC care, but were given 2 kits to have the option of testing as a couple with their male partner. The women were interviewed 3 months after the baseline interview to assess the status of HIV testing for the male partner since the baseline interview, and the method of testing (e.g., using the self-testing kit or testing at a clinic). Women were also asked about perpetration of IPV from the male partners during the study, but no IPV was reported. The male partners were also contacted at 3 months after the female baseline interview, and they were administered a questionnaire including variables from both the female baseline and the female 3-month follow-up surveys if they consented for an interview. For this analysis, only the data from Arm 3 (the intervention arm) were used. At the time of the original trial, HST kits were not yet approved for use in Kenya, so the only way to acquire these kits was through participation in the RCT. Therefore, because the primary outcome in this current analysis was the use of HST kits, we limited the analysis to participants in the intervention arm, because participants in the control arms had no way of acquiring these kits.

### Measurements

The 2 primary exposure variables used in this study are 2 markers of gender equality—decision making power as assessed by the female, and attitudes toward IPV as reported by the male's personal attitudes. Decision making power was measured by the woman's report for the validated Household Decision Making Scale, a 3-item scale regarding the woman's decision making in 3 areas: visiting family or relatives, major household purchases, and daily household needs.^24^ These variables had available selections of the female partner alone, her male partner or someone else alone, or jointly between the female and male partner. During data analysis, each response to the 3 questions was dichotomized, and took on a value of 0 if the decision was made by her male partner or someone else, and a value of 1 if the woman reported that the decision was made by either herself or jointly with her male partner. An index was created by summing those 3 dichotomized responses to assess the level of decision making power by the female partner. This index took on a value of 0 if the woman made no decisions by herself or jointly (no decision making power), 1 if she made 1 or 2 decisions by herself or jointly (low decision making power), and 2 if she made all 3 decisions by herself or jointly (high decision making power). Attitudes toward IPV was measured by the male partner's report for the validated Violence Domain of the Gender Equitable Men Scale, a 5-question scale regarding hypothetical violence toward women, with answers of either agree (score of 1) or disagree (score of 3).^25^ An index was created by summing scores across all 5 questions, and was categorized into 3 levels: high acceptance of IPV (score of 5–11), medium acceptance of IPV (score of 13), and low acceptance of IPV (score of 15), where the higher the score, the lower acceptance of IPV, and therefore higher support for equitable gender norms.^25^ The primary outcome variable was couples' uptake of the HST kit, as assessed by the combined reports of the woman's and man's response. This binary variable took on 2 values: either couples' tested together using the HST kits, or they did not (which included either testing together by other means or not testing together).

Covariates included age of both the man and woman (categorized from a continuous variable based on distributional balance), education (primary or lower, or secondary or higher), employment status (self-employed, employed for wages, or other), marital status (currently married or not currently married), previous HIV testing by the woman (tested for HIV before or had not), male partner's alcohol and drug use (currently using or not currently using), equality in earnings (the proportion of household expenses met by the woman's earnings: none, less than a third, a third to a half, and more than half), and wealth index (a composite measure of a household's cumulative living standard constructed by the International Demographic and Health Surveys Program).^26^ The wealth index consisted of the following variables: source of drinking water for the household, type of toilet facility for the household, sharing of toilet with other households, type of fuel used for cooking, ownership of transportation (bicycle, motorcycle, car), any modern appliances in the home (electricity, solar panels, generator, radio, television, refrigerator, telephone), material of the house floor and roof, ownership of land or their house, ownership of any productive assets (e.g., cattle or a sewing machine), and cash savings. Rasch modeling was performed in the original trial to create the wealth index, and then was separated into quartiles.^23^ Many of these covariates were found to be associated with the 2 primary exposures of attitudes toward IPV and decision making power in our previously published analyses, so they were included in this study.^27^

### Data Analysis

SAS 9.4 (SAS Institute, Cary, NC) was used for all analyses. Descriptive statistics were conducted with mean and SD (for continuous variables), and proportions (for categorical variables). Cochran Mantel-Haenzel or Cochran-Armitage Trend tests were used for comparisons in bivariate analyses. Modeling was performed with a generalized linear mixed models framework to account for clinic site-level clustering.^28^ All analyses included a binary variable of couples' uptake of the HST kits, as assessed by the combined reports of the woman's and man's response as the primary outcome. The first set of analyses focused on gender equality as measured by attitudes toward IPV from the man's report, and the second set of analyses focused on gender equality as measured by decision making power from the woman's report as the primary exposure. Generalized linear mixed model was used to estimate odds ratio (OR) and corresponding 95% confidence interval (CI) for both sets of analyses. We ran sequential modeling for each set of analyses, first running unadjusted analysis, and then added sets of domains (demographic variables and economic variables, and then all of the previous variables and behavioral variables). Two measures of model fit were used to assess confounding and potential modification (−2 Log Likelihood and *R*^2^ values, when appropriate). The 95% CI not including 1 was used for significance for our primary exposures.

### Ethical Approval

The original trial was approved by the institutional review board of the Kenya Research Medical Institute (IRB no. 485). Written informed consent was obtained from all participants. The current data analysis was performed on completely deidentified data and was deemed by the institutional review board of the Medical University of South Carolina to not be human subjects research.

## RESULTS

Table 1 shows the demographic characteristics of the women and their male partners. Overall, 1,410 women were enrolled and randomized into the study, with 472 women enrolled and randomized into the intervention arm (with the provision of the HST kits), and 422 women were interviewed at the 3-month follow-up visit. The original study attempted to reach all 472 male partners in the intervention arm, and 395 male partners were interviewed at the 3-month follow-up visit. Male partners were on average older than the women (31.7 years versus 26.7 years, respectively), and in 83.9% of the relationships, the man was older than the woman. For women, the majority had a primary or lower education (52.3%), were mostly Protestant or other Christian besides Catholic (76.1%), were mostly self-employed (51.7%), were currently married (86.9%), had less than a third or none of the household expenses met by their earnings (64.2%), and the vast majority were HIV negative (96.6%). For the men, the majority had a secondary or higher education (66.2%), were mostly Protestant or other Christian besides Catholic (68.6%), were either employed for wages or self-employed (47.9% and 47.3%, respectively), were currently married (89.4%), and the vast majority were HIV negative (98.3%). The variables that were significantly different between male and female partners were age, education, religion, and employment. Overall, 19.6% of the men showed high acceptance of hypothetical IPV, 21.2% had moderate acceptance of IPV, and 58.7% had low acceptance of IPV. For decision making power, 12.7% of the women had no decision making power, 31.1% of the women had low decision making power, and 56.1% had high decision making power. Overall, 81% of the couples tested together using the HST kits.

**TABLE 1:**
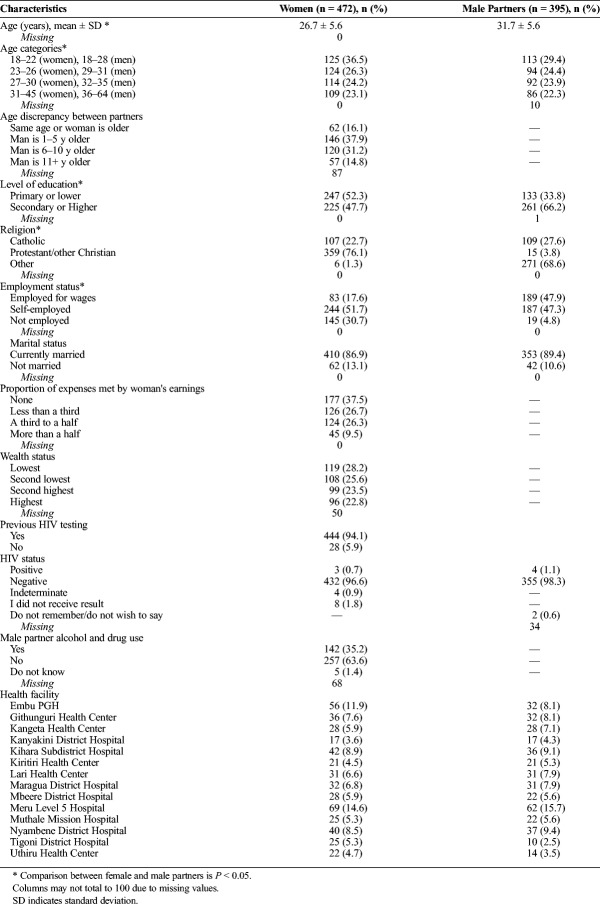
Characteristics of Women Attending Antenatal Care at Baseline and Characteristics of Male Partners at Month 3 in Central Kenya

Table 2 shows the bivariate association between the primary exposures and couples' uptake of HST. The Gender Equitable Man Scale was significantly associated with couples' uptake of the HST kits, showing that lower acceptance of IPV was associated with higher couples' HST uptake (*P* < 0.01).

**TABLE 2:**
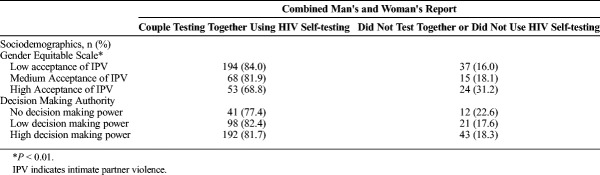
Bivariate Analysis of Gender Equality and Couples HIV Self-Testing Uptake

Table 3 shows the modeling of couples' uptake of HST kits by gender equality. With gender equality measured as attitudes toward IPV, the unadjusted models found that those with medium acceptance of IPV and low acceptance of IPV had more than double the odds of using the HST kits to test as a couple as compared with those with high acceptance of IPV (OR, 2.27; 95% CI, 1.03–4.99, and OR, 2.27; 95% CI, 1.37–5.17, respectively). Adjusting the model for age, male and female education, male employment, marital status, wealth status, previous female HIV testing, and male partner alcohol/drug use showed that those with medium acceptance of IPV still had higher odds of using couples HST (although no longer statistically significant) compared to those with high acceptance of IPV (OR, 2.36; 95% CI, 0.99–5.63). Those with low acceptance of IPV had 2.5 times the odds of using couples' HST compared with those with high acceptance of IPV (OR, 2.50; 95% CI, 1.20–5.21). We did not find any statistically significant results for the association between decision making power (with an index of decision making regarding major household purchases, daily household needs, and visiting family) and couples' uptake of HST, both with unadjusted and adjusted models.

**TABLE 3:**
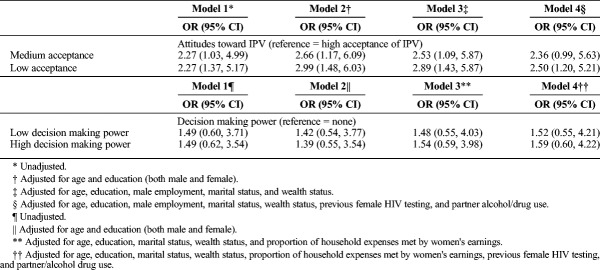
Modeling of Associations between Gender Equality and Couples HIV Self-Testing Uptake

## DISCUSSION

In this study, we examined uptake of HST among heterosexual couples expecting a child in Kenya, where the pregnant women brought home 2 oral self-testing kits from ANC to present to her male partner for HIV testing. This study was conducted to examine the association between gender equality (as measured by male partner's attitudes toward IPV, and woman's report of decision making power) and uptake of HST uptake among these couples. The decision making power index was not significantly associated with couples' uptake of HST. As the decision making power index consisted of power regarding major household purchases, daily household needs, and visiting family, there could be a mixing of effects within the index due to the differing proportions within the component variables (67% woman-only or joint decisions for major household purchases, 80% woman-only or joint decisions for daily household needs, and 72% woman-only or joint decisions for visiting family). However, among couples where the man had low acceptance of IPV, there was 2.5 times higher odds of couples' uptake of HST compared with couples where the man had high acceptance of IPV.

Eighty-one percent of the participants tested together as a couple using the HST kits. This high proportion underscores the promise of HST to increase testing rates, and corroborates other studies showing high acceptability of HST and high uptake of this testing method,^13,14^ including among male partners of pregnant women.^29^ In the parent RCT of these analyses, men in the intervention arm had much higher uptake of testing in general compared with the 2 control arms (79.4% vs. 37% and 28%, respectively), and in the intervention arm, 95% of the men who tested for HIV used the HST kit.^23^ The Joint United Nations Programme on HIV/AIDS (UNAIDS) put forth a target that states by 2020, 90% of people living with HIV should know their status, 90% of people with diagnosed HIV should receive antiretroviral therapy, and 90% of people on antiretroviral therapy should be virally suppressed.^30^ The HST seems to be an important way to contribute toward reaching that first 90% goal. Future research should examine the potential differences in HST uptake between more stable relationships, like these heterosexual primary partners expecting a child, versus more casual sexual relationships. These results show the benefits of appropriate attitudes regarding IPV on couples testing together using this new testing technology of HST. Our results suggest that if the male partner does not accept IPV, he may be more likely to be open for discussion within the partnership, and more willing to test for HIV with their female partner. Male partners less accepting of IPV may be more accepting of the scenario in which the pregnant female partner brings home self-testing kits from the clinic and initiates the discussion about HIV testing. If these individuals are more willing to test for HIV as a couple using these self-testing kits, and they do test positive, this could have important implications in reducing transmission of HIV between heterosexual partners in a relationship, and prevention of mother-to-child transmission of HIV. Second, these findings highlight a potential dual intervention. A community-based HST study in Malawi found that fear of HIV discordant test results, unequal household gender roles, and couple dynamics were barriers for couples to self-test together.^31^ It is possible that an intervention focused on reducing men's acceptance of IPV, could also have the dual benefit of increasing the men's willingness to self-test for HIV, especially with a sexual partner. A community-level intervention trial in Uganda attempting to shift harmful social norms that promote gender inequality found that males in the intervention group, over a 1-year follow-up, were more likely to have an HIV test compared with controls.^32^ Furthermore, qualitative interviews with men participating in a rights-based gender equality and health program intervention in South Africa found that men who participated reported an increased capacity to overcome masculinity-related barriers to HIV testing, and had increased ability to discuss HIV with others, which led to greater willingness to be tested for HIV.^33^ These interventions dealt with standard HIV testing, but future research in this area could potentially confirm these results with couples' HST uptake as well.

There are several limitations in this study. This study population might be limited in the generalizability of the results, as this analysis was limited to heterosexual couples expecting a child, and women self-excluded from the original trial if they were concerned about IPV. Furthermore, IPV concerns or negative results when offering self-testing could be different among the participants who were lost to follow-up, for which we have no data. However, the original trial had very high participation rates with very few women self-excluding due to IPV concerns. There is also a limitation in the measurement of gender equality within this data. Gender equality cannot be generalized beyond how it is measured. In this study, gender equality was measured as attitudes toward IPV, and decision making power was measured by decision making regarding visiting family, major household purchases, and daily household needs. There could be other ways of measuring gender equality that were not captured in this analysis, including influences on HIV preventive behaviors like condom use or measures of relationship quality.

In summary, lower acceptance of IPV from the male partner of pregnant women in central Kenya is significantly associated with more than double the odds of HST as a couple compared with couples in which the men had high acceptance of IPV. This study appears to be the first to investigate the relationship between gender equality and uptake of HST. Realizing the importance of low acceptance of IPV in increasing couples testing, especially in the context of HST, is vital as we work toward achieving the first 90% in the UNAIDS 90:90:90 target.
